# Non-rodent Models of Atherosclerosis: Repurposing of Existing Drugs and Search for Novel Treatment Strategies

**DOI:** 10.2174/011573403X316529240919103119

**Published:** 2024-10-03

**Authors:** Siarhei A. Dabravolski, Victoria A. Khotina, Mikhail A. Popov, Victor Y. Glanz, Vasily N. Sukhorukov, Alexander N. Orekhov

**Affiliations:** 1 Department of Biotechnology Engineering, Braude Academic College of Engineering, Snunit 51, P.O. Box 78, Karmiel 2161002, Israel;; 2 Petrovsky National Research Center of Surgery, Abrikosovsky lane, 2, 119991 Moscow, Russia;; 3 Institute of General Pathology and Pathophysiology, 8 Baltiyskaya Street, Moscow 125315, Russia

**Keywords:** Atherosclerosis, animal models, immunotherapy, chronic inflammation, cardiovascular diseases, pathology

## Abstract

Atherosclerosis and associated cardiovascular diseases are the leading causes of illness and mortality worldwide. The development of atherosclerosis is a complex process involving oxidative stress, surplus lipid deposition and retention, endothelial dysfunction, and chronic inflammation. Developing novel anti-atherogenic and repurposing existing drugs requires the use of suitable animal models to characterise the fundamental mechanisms underlying atherosclerosis initiation and progression and to evaluate potential therapeutic effects. Commonly used rodent models, however, are not always appropriate, and other models may be required to translate these discoveries into valuable preventive and treatment agents for human applications. Recent advances in gene-editing tools for large animals have allowed the creation of animals that develop atherosclerosis faster and more similarly to humans in terms of lesion localisation and histopathology. In this review, we discuss the major advantages and drawbacks of the main non-rodent animal models of atherosclerosis, particularly rabbits, pigs, zebrafish, and non-human primates. Moreover, we review the application of recently invented novel therapeutic methods and agents, and repurposed existing drugs (such as antidiabetic and anticancer) for atherosclerosis treatment, the efficacy of which is verified on non-rodent animal models of atherosclerosis. In total, the proper selection of a suitable animal model of atherosclerosis facilitates reproducible and rigorous translational research in repurposing of existing drugs, discovering new therapeutic strategies, and validating novel anti-atherosclerotic drugs.

## 1 Introduction

Cardiovascular diseases (CVD) are the major cause of morbidity and mortality worldwide, with atherosclerosis being its primary underlying pathology [1]. Atherosclerosis is a multi-faceted chronic inflammatory disease affecting major blood vessels through plaque-forming focal retention and aggregation of lipid materials in the arterial wall [2–4]. The progression of atherosclerosis is marked by the gradual narrowing and solidification of the arterial lumen, which, subsequently, compromises blood flow and resulting in angina [5]. In advanced stages of atherosclerosis, the plaque may rupture and lead to atherothrombotic vessel occlusion and further life-threatening severe cardiovascular events, such as ischaemic stroke and myocardial infarction [6].

**Fig. (1):**
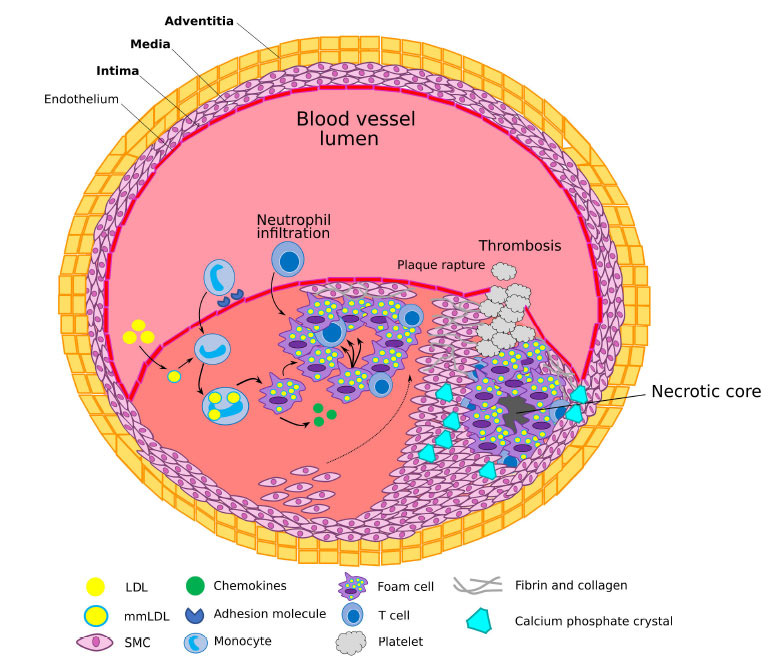
Atherosclerosis initiation and progression. Different adverse factors (such as hyperglycaemia and oxidative stress) initiate the development of atherosclerosis. Low-density lipoprotein (LDL) particles accumulate in the intima, where they can undergo various modifications to form multiple modified forms of LDL (mmLDL), which can provide them with pro-inflammatory and immunogenic properties. Activated endothelial cells (EC) express adhesion molecules which bind monocytes, and chemokines further promote migration of the bound monocytes into the bloodstream. Monocytes maturate into macrophages, bind and engulf LDL particles and transform into foam cells. Less abundant T cells also enter the intima and regulate functions of EC, smooth muscle cells (SMC) and innate immune cells. SMC migrate to towards the injured area and proliferate, generating the atherosclerotic plaque structure, which limits the blood flow and nutrient supply to surrounding tissues. Apoptosis and suppressed efferocytosis inside the lipid core (depicted with triple arrows over the foam cell inside the lipid core) causes the secondary inflammation and necrosis, resulting in formation of the necrotic core. Calcium homeostasis dysregulation and impaired clearance in the plaque activates a mineralisation process, resulting in the formation of calcium phosphate crystals. The spotty type of calcification usually increases plaques instability, while extended calcification can stabilise plaques. Plaque rupture is the common complication of atherosclerosis in advanced stages; it activates thrombotic events which can completely block the blood flow and cause myocardial infarction or stroke.

Currently, the elevated level of low-density lipoprotein (LDL) in plasma is recognised as the primary biological risk factor contributing to atherosclerosis initiation [7]. Furthermore, within such a pathological microenvironment, modified proteoglycans with elongated glycosaminoglycan chains and increased levels of reactive oxygen species (ROS) facilitate the formation of immune-reactive multiple modified forms of LDL (mmLDL) (*e.g*., glycation, desialylation, oxidation, dicarbonyl, and others) [8-11]. MmLDL promotes oxidative stress and an inflammatory response in endothelial cells (EC), inducing the expression of cytokines such as (*tumour necrosis factor α* (*TNFα*), Interleukins (*IL-1β*, *IL-2*, *IL-6*, *IL-8*, *IL-12*, *IL-17*), chemokines such as *chemokine (C-X-C motif) ligand 1* (*CXCL1*), *CXCL2*, *CXCL10* and *monocyte chemoattractant protein-1* (*MCP-1*), adhesion molecules such as *Intercellular adhesion molecule 1* (*ICAM-1*), *Vascular cell adhesion protein 1* (*VCAM-1*), *E-selectin*, and *P-selectin*) and enzymes such as *Cyclooxygenase* (*COX*) and *Inducible nitric oxide synthase* (*iNOS*), which facilitate the recruitment of monocytes through the endothelium into the arterial wall, where they differentiate into macrophages, engulf oxLDL, and transform into foam cells, a hallmark of atherosclerotic plaques (Fig. **1**) [12, 13]. Foam cells secrete numerous mediators of inflammation, which further promote monocyte recruitment, resulting in the proliferation and migration of vascular smooth muscle cells (VSMC) from the tunica media into the sub-endothelial space. In the sub-endothelial space, VSMC secrete extracellular matrix proteins, thus contributing to plaque expansion [14].

Advanced human plaques exhibit distinct features, such as a large necrotic core, surplus lipid-laden and activated macrophages, a low level of VSMC and derived collagen, defective efferocytosis and upregulated inflammatory response (Fig. **1**) [15, 16]. Further plaque progression is characterised by the formation of a thin fibrous cap, appearance of haemorrhages and neovascularisation within the plaque. Matrix metalloproteinases (MMP) and their inhibitors play a complex role in the advanced stages of atherosclerotic plaque progression and rupture [17]. The rupture of such plaques results in intra-luminal thrombosis, which may cause sudden arterial blockage and cessation of blood flow, leading to life-threatening cardiovascular events such as a myocardial infarction, stroke, and even sudden death [18]. Additionally, the deposition of calcium ion-containing complexes in vessel walls, valves, and plaques greatly affects blood circulation and plaque stability and may lead to death (Fig. **1**) [19, 20]. Understanding the complexity of atherosclerotic lesions and their clinical manifestations requires further research to enhance our understanding of the underlying molecular mechanisms and to identify effective therapeutic interventions [21-23].

## 2 ANIMAL MODELS IN ATHEROSCLEROSIS RESEARCH

The animal models serve two major purposes – to increase our understanding of the intricate molecular mechanisms underlying atherosclerotic plaque formation, development, and rupture and associated cardiovascular events and to aid the development and evaluation of innovative anti-atherosclerotic drugs [24]. There are several standard ways to induce atherosclerosis in animal models: 1) apply a specific diet (cholesterol/fat-rich); 2) manipulate cholesterol metabolism-related genes [25]; 3) use specific chemicals to interrupt normal lipid metabolism [26, 27]; 4) surgical interventions [28]; or 5) introduce other risk factors associated with atherosclerosis (such as diabetes or hypertension) [29-31]. The development of atherosclerosis in humans takes many years, with the formation of an initial lesion during adolescence and the appearance of severe complications at the advanced age of 50-60. For research purposes, however, it is crucial to obtain progressive atherosclerotic lesions within a reasonable time frame, thus development of atherosclerosis in animal models should be greatly accelerated compared to the human course of atherosclerosis. Therefore, the ideal animal model should closely mirror human anatomy and pathophysiology, be readily available, cost-effective, easy to maintain and handle, and conducive to medical and pharmaceutical research.

Currently, rodent models, particularly mice, dominate atherosclerosis academic research and in the testing of novel pharmacotherapies [32, 33]. However, despite the generated atherosclerotic lesions having a high degree of morphological similarity to human lesions, the spatial distribution of these plaques often differs from that observed in humans [34]. Another concern is that these monogenic atherosclerosis models might not encompass all essential processes involved in multifactorial lesion formation in humans [35] and do not fully recapitulate the severe clinical complications stemming from plaque rupture and erosion (such as myocardial infarction and stroke) [36].

**Fig. (2):**
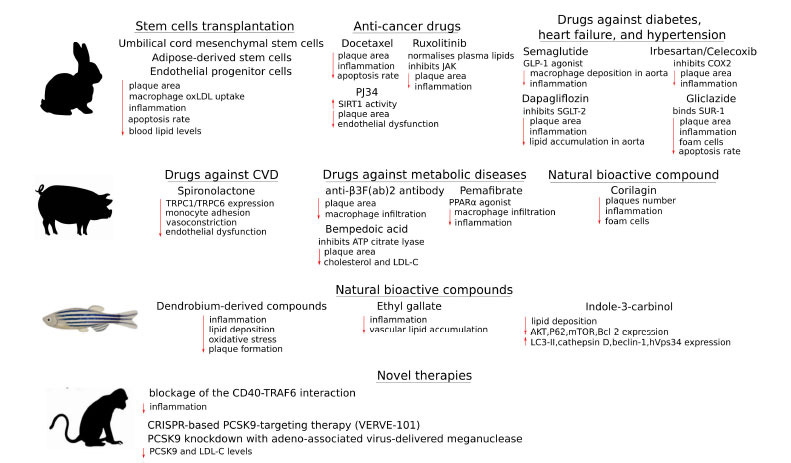
The summary of novel therapies and various drugs re-purposed to treat atherosclerosis. The major effects of treatment are depicted with up- or down-ward red arrow. Oxidised LDL - OxLDL; Janus kinase - JAK; NAD-dependent deacetylase sirtuin-1 - SIRT1; Glucagon-like peptide-1 - GLP-1; Sodium/glucose cotransporter 2 - SGLT-2; Cyclooxygenase-2 - COX2; Sulfonylurea receptor 1 - SUR-1; Transient receptor potential canonical 1 - TRPC1; Peroxisome proliferator-activated receptor α - PPARα; Proprotein convertase subtilisin/kexin type 9 - PCSK9.

Despite the prevalence of mouse models, researchers are increasingly turning to other non-rodent animals to unravel the complexity of atherosclerosis development and to explore potential pharmacotherapies for the prevention and treatment of atherosclerosis. Such models include rabbits, birds, pigs, non-human primates (NHP), and even zebrafish [24,37]. Additionally, the invention and constant improvement of novel gene editing techniques and methods to create transgenic animals (such as CRISPR/Cas9 systems, transcription activator-like effector nuclease (TALEN), and zinc finger nuclease (ZFN) have greatly expanded the spectrum of available animal models [38,39]. Further, in this review we discuss the results of recent research focused on discovering and validating novel anti-atherosclerotic drugs and treatment methods, repurposed drugs, and natural bioactive compounds on non-rodent models of atherosclerosis (in particular, rabbit, pig, and zebrafish as well as non-human primates) (Fig. **2**). These studies aim to improve our understanding of the pathological mechanisms and clinical repercussions of atherosclerosis in humans and enhance existing approaches to preventing and treating atherosclerosis.

### 2.1 Rabbit Models

New Zealand white rabbits were the major subjects for experimental atherosclerosis even before the transgenic mouse model emerged [40]. Subsequently, selective breeding of Watanabe heritable hyperlipidemic (WHHL) rabbits, an animal model of familial hypercholesterolaemia, led to the development of other strains that exhibit spontaneous coronary atherosclerosis (WHHL-CA) or myocardial infarction (WHHLMI) [41, 42]. St. Thomas' mixed hyperlipidaemic (SMHL) rabbit, a model of familial combined hyperlipidaemia (FCH), is another well-studied strain critical for atherosclerosis research [43]. Advancements in gene editing techniques, particularly zinc finger nuclease, helped to create more advanced genetic models [44, 45], which include also models of atherosclerosis, such as *APOE*, *APOC3* and *CETP* knockout (KO) rabbits [46-48]. Accordingly, *APOE* KO rabbits showed elevated cholesterol and triglyceride (TG) levels on a standard diet [46], while *APOC3* KO rabbits demonstrated accelerated catabolism of TG-rich lipoproteins, which resulted in resistance to cholesterol-rich diet-induced hyperlipidaemia and inhibited atherosclerosis [47, 49], and *CETP* KO rabbits even on cholesterol-rich diet had higher HDL-C levels and lower total cholesterol levels, and reduced levels of both aortic and coronary atherosclerosis compared to wild-type rabbits [48].

The lipid metabolism system of rabbits and humans is rather similar, which facilitates the use of rabbits to study human lipid metabolism. Rabbits, unlike humans, exhibit atherosclerosis more prominently in the aortic arch and descending thoracic aorta, rather than in the abdominal aorta [50]. Comparative transcriptome analysis of mouse and rabbit models under commonly used atherogenic conditions demonstrated that APOB and APOA4, respectively, were the major lipoprotein metabolism responding genes. Moreover, the rabbit model was more similar to developing human atherosclerosis when considering the expression change of *APOA4* and *APOB*, thus making the rabbit a more suitable animal model to investigate human atherosclerosis [51]. Additionally, lesions in rabbits often consist of large collections of foam cells, which, with the help of genetic or mechanical manipulation, can progress to more complex stages, including symptomatic and thrombotic lesions in the coronary arteries [52]. In comparison to the rodent, the size of the rabbit aorta allows for catheter-based procedures, preclinical studies for surgical experiments, and non-invasive imaging using clinical equipment, although the coronary arteries are too small. As a disadvantage of rabbits as a model system, it is important to mention their higher individual and keeping price, breeding cost, and requirement for a much larger space [45].

#### 2.1.1 Novel Methods of Atherosclerosis Treatment Explored with Rabbit Models of Atherosclerosis

##### 2.1.1.1 Stem/Progenitor Cells Transplantation

Currently, stem cells are considered a promising research area for the effective treatment of atherosclerosis [53]. Recently, human umbilical cord mesenchymal stem cells (UCSC) have been successfully applied to prevent atherosclerotic plaque formation and lesion progression in high-fat diet-fed rabbit models (Fig. **2**). In particular, UCSC injection resulted in a reduction of the aortic plaque area and decreased the levels of pro-inflammatory cytokines (IL-6 and TNFα), while the levels of anti-inflammatory cytokines (IL-10 and TGFβ) in the aorta atherosclerotic plaque were increased. Additionally, macrophage accumulation, apoptosis rate, and macrophage oxLDL uptake were decreased, and the expression of macrophage scavenger receptors *CD36* and *SRA1* was decreased after UCSC treatment, suggesting that UCSC transplantation may be an effective strategy for atherosclerosis treatment at the early and more progressive stages of plaque formation [54].

Similarly, intravenous transplantation of allogeneic rabbit adipose-derived stem cells (ADSC) was investigated in a high-fat diet rabbit atherosclerosis model both *in vitro* and *in vivo*. *In vitro*, ADSC facilitated macrophage switch towards an M2 anti-inflammatory phenotype, increased IL-10 and decreased TNFα secretion, increased *arginase 1*, and decreased *inducible nitric oxide synthase* (*iNOS*) expression. When applied *in vivo*, ADSC reduced blood lipid levels, plaque area, macrophage oxLDL uptake, and the expression of *CD36*, *SRA1*, *IL-6,* and *TNFα*. On the contrary, the expression levels of anti-inflammatory *IL-10* and *TGFβ* were increased, confirming the high therapeutic potential of allogeneic rabbit ADSC transplantation [55].

Endothelial progenitor cells (EPCS) represent another effective therapeutic method. Thus, transplantation of bone marrow-derived EPCS expressing human *dimethylarginine dimethylaminohydrolase* (*DDAH*), a gene crucial for NOS regulation and endothelium dysfunction, to an atherosclerotic rabbit model provided anti-proliferative and anti-atherogenic effects, reduced plaque size and increased endothelium integrity and repair compared to the control group. Moreover, the expression of *lectin-type oxidised LDL receptor 1* (*LOX-1*), adhesion molecule *vascular cell adhesion molecule 1* (VCAM1), and chemokine *monocyte chemoattractant protein-1* (*MCP1*) was decreased in the experimental group [56]. These results suggested that transplantation of stem/progenitor cells is a valuable tool for the treatment of atherosclerosis by improving endothelial cell function and providing anti-inflammatory, anti-proliferative, and anti-atherogenic effects. Moreover, genetic engineering may provide an additional option to increase the efficiency of this therapeutic strategy by adapting it for certain cardiovascular complications.

##### 2.1.1.2 Drug repurposing

Drug repurposing is an important recent trend as drugs originally designed to treat one disease have been successfully applied for the treatment of other diseases. In the case of cardiovascular disease, the repurposed drugs are mostly antidiabetic because they target similar metabolic pathways [57].

###### 2.1.1.2.1 Anti-Cancer Drugs

Moreover, other types of drugs have also been used to treat atherosclerosis. Thus, docetaxel (DTX) is a well-known chemotherapeutic agent with a long history of usage against various types of cancers, which, despite constant improvement, still has numerous serious drawbacks and side effects [58]. As it was shown, the application of low doses of DTX allowed to avoid major side effects but provided a significant anti-atherosclerotic effect [59]. In particular, intravenous injection of DTX carried in nanoparticles to atherosclerotic rabbits greatly reduced the atheroma area while showing no renal, hepatic, or haematological toxicity (Fig. **2**). Moreover, the macrophage expression of *CD36*, inflammation- (*NF-κB*, *TNFα*, *IL-1β*, *IL-6),* and adhesion- (*MCP1* and *von Willebrand factor*) related genes was reduced. The levels of pro-apoptotic (caspase 3/9 and Apoptosis regulator BAX), matrix metalloproteinases (MMP-2/9) and collagen 1/3 proteins were reduced with DTX treatment. However, the levels of anti-apoptotic B-cell lymphoma 2 (Bcl-2) and anti-inflammatory TGFβ proteins were also reduced by DTX treatment, which suggested that the demonstrated effects on lesion inflammation and proliferation processes require further investigation. In conclusion, considering the absence of toxicity and significant beneficial effects, DTX could be considered a candidate for future clinical trials as an anti-atherosclerosis drug [60].

PJ34, the inhibitor of Poly [ADP-ribose] polymerase 1 (PARP-1), which is involved in the regulation of cell differentiation and proliferation, recovery from DNA damage, induction of inflammation, and pathophysiology of type I diabetes [61], was recently proposed as a potential drug against cancer [62], endothelial dysfunction and hypertension [63]. Recent research has demonstrated the anti-atherogenic potential of PJ34, where PJ34 treatment protected EPCS derived from atherogenic rabbits from H_2_O_2_-mediated oxidative stress by promoting SIRT1 activity and preserving intracellular NAD+ levels. *In vivo,* PJ34 application reduced atherosclerotic plaque area and prevented endothelial dysfunction in atherosclerotic rabbits [64].

Ruxolitinib is a Janus kinase (JAK) inhibitor, which interrupts JAK/signal transducer and activator of transcription (STAT) signalling pathway, mostly in lymphocytes. Currently used against various inflammatory diseases and some types of cancer (for example, myelofibrosis - a rare type of blood cancer) [65, 66], ruxolitinib reduced the area of atherosclerotic plaques in rabbits fed with high-fat diet and subjected to balloon injury of the aorta. Ruxolitinib improved plasma lipid parameters in atherosclerotic rabbits (increased level of HDL-C and decreased levels of TC, TG, and LDL-C). Moreover, the levels of pro-inflammatory markers (IL-1β, IL-6, TNFα, and IFNγ) were decreased, while the levels of anti-inflammatory markers (IL-10 and IL-17) were increased in the plasma of atherosclerotic rabbits. These results suggest that the JAK inhibitor ruxolitinib may be a promising drug for the treatment of atherosclerosis [67].

###### 2.1.1.2.2 Drugs Against Diabetes, Heart Failure, and Hypertension

Irbesartan, an angiotensin II receptor antagonist widely used for the treatment of high blood pressure, heart failure, diabetic kidney disease, and some other conditions [68], has demonstrated anti-atherosclerotic effects alone and in combination with celecoxib, a nonsteroidal anti-inflammatory drug acting through COX2 inhibition [69]. Treatment of atherosclerotic rabbits with irbesartan and celecoxib reduced the surface area of aortic atherosclerotic lesions and lowered the expression levels of *COX-2* and *MMP-9*, as well as NF-κB activity, thus confirming their anti-atherosclerotic effects on aortic plaques [70].

Semaglutide is a peptide long-acting glucagon-like peptide-1 receptor agonist used as a medication for long-term weight management and treatment for type 2 diabetes and obesity [71]. Based on multi-modality positron emission tomography and computed tomography analysis, atherosclerotic rabbits treated with semaglutide demonstrated reduced macrophage deposition in the aorta cross-sections, decreased inflammatory macrophage activity, and vascular inflammation. However, no difference in micro-calcification parameters was found between semaglutide and control groups [72]. Nano-encapsulated gliclazide, another drug against type 2 diabetes [73], also reduced plaque lesion area, levels of total foam cells and lipid-laden macrophages in aorta sections of atherosclerotic rabbits. *In vitro* treatment of LPS-induced monocytes with gliclazide, a selective interactor for Sulfonylurea receptor 1 (SUR-1), reduced the expression of apoptosis and inflammation-related genes (such as *IL-1β*, *IL-18*, *NLRP3*, *NOS*, *MyD88* and *caspases 1/3/8/9*), thus suggesting that anti-atherogenic properties of gliclazide are based on the profound anti-inflammatory effect [74].

Dapagliflozin, a sodium-glucose cotransporter-2 (SGLT-2) inhibitor used to treat type 2 diabetes, chronic kidney disease, and heart failure [75-77], also demonstrated similar effects (Fig. **2**). Treatment of atherosclerotic rabbits with dapagliflozin resulted in lower stenosis diameter, reduced lipid accumulation in the aorta, and decreased macrophage infiltration within the plaques, while the level of M2 phenotype macrophages was higher. Moreover, the plaque levels of IL-1β, IL-6, and TNFα were lower in the dapagliflozin group. Experiments *in vitro* showed that dapagliflozin attenuated inflammatory responses in macrophages by reducing the expression of inflammatory mediators (such as *Toll-like receptor 4* (*TLR4*), *NF-κB*, *IL-6* and *TNFα*) [78].

### 2.2 Pig Models

Pigs represent large animal models with a size comparable to humans, similar cardiovascular anatomy, and genetic traits compared to rodents, along with easier housing and handling compared to non-human primates. While there are some genetic differences, such as the absence of CETP and lipoprotein(a) (Lp(a)), pigs offer a favourable balance between human-like physiology, convenience in housing and handling, and the accessibility of research tools for various essential inquiries in atherosclerosis research. Despite pigs being prone to develop diet-induced atherosclerosis, they often require a combined diet of high dietary cholesterol coupled with cholic acid. However, even on an atherosclerosis-stimulating diet, disease progression beyond foam cell formation may require significant time [79]. To overcome these limitations, various approaches (such as mechanical damage to arteries, selection for natural genetic mutations, introduction of atherosclerosis risk factors, and genetic engineering) can be used [80, 81].

The groundbreaking achievement in identifying natural mutations in the *apolipoprotein B* and *LDL receptor* (*LDLR*) genes has significantly reduced the time required for atherosclerosis development, thereby increasing the usability of the porcine model of atherosclerosis [82]. Despite bearing a striking resemblance to human lesions, these pigs typically weigh over 200 kg, making them impractical and costly to work with. However, through a series of breeding efforts with smaller pig strains, a much smaller hypercholesterolaemic pig strain named familial hypercholesterolaemia Bretoncelles Meishan (FBM) pigs was created [83]. Later, genetic engineering techniques were used to knock out the *LDLR* gene in Yucatan minipigs, resulting in fully *LDLR*-deficient animals that displayed high susceptibility to diet-induced hypercholesterolaemia and developed atherosclerotic lesions in several months [80].

Atherosclerotic lesions in pigs share some pathological features with human lesions: 1) they tend to localise in the proximal segments of coronary arteries, iliofemoral artery and abdominal aorta; 2) plaque neovascularisation and intraplaque haemorrhages are common and abundant; 3) both calcification granules and solid calcifications are frequent within and around necrotic cores. However, thrombotic complications in porcine models of atherosclerosis are rare [79,80]. In total, with histopathological characteristics closer to human lesions and a human-like body size enabling the use of clinical equipment without modifications, the porcine model of atherosclerosis offers several advantages in addressing specific challenges in atherosclerosis compared to rodent and rabbit models. It plays an increasingly vital role in tackling issues that currently hinder our comprehension, diagnosis, and treatment of human atherosclerosis. Further, in this section, we review recent research conducted on pig models for testing new and repurposed existing drugs for the treatment of atherosclerosis.

#### 2.2.1 Drug Repurposing Explored on Pig Models of Atherosclerosis

##### 2.2.1.1 Drugs Against Heart Failure and Hypertension

Spironolactone, a mineralocorticoid receptor inhibitor, is commonly used to treat various cardiovascular diseases, including heart failure and hypertension [84, 85]. It attenuated atherosclerosis and endothelial dysfunction in metabolic syndrome (MetS) pigs (castrated Ossabaw miniature pigs) (Fig. **2**). The beneficial effects of spironolactone were mediated by regulating the coronary *transient receptor potential canonical 1* (*TRPC1*) channel expression. Thus, *TRPC6* was expressed at a low level in atheromas while at a high level in the medial layer of MetS pig coronary arteries compared to that in lean pigs. On the contrary, higher expression of *TRPC1* was detected in macrophages localised in MetS atheromas, and was weak in the medial layer. Mechanistically, spironolactone counteracted the aldosterone-induced rise in monocyte adhesion in coronary arteries of the pigs by decreasing the expression of *TRPC1* and *TRPC6* channels in coronary arteries, reducing MetS-associated atherosclerosis, endothelial dysfunction and vasoconstriction [86].

##### 2.2.1.2 Drugs Against Metabolic and Inflammatory Diseases

Diabetes is one of the major risk factors facilitating atherosclerosis development. Hyperglycaemia triggers vascular cells to release extracellular matrix proteins that bind to the αVβ3 integrin, which promotes VSMC migration and proliferation, thus stimulating atherosclerosis [87]. Thus, treatment of diabetic (induced with streptozotocin) Yorkshire male pigs fed on a high-fat diet (cholesterol + cholic acid) with anti-β3F(ab)2 antibody reduced macrophage level within lesions and decreased lesion size in the coronary arteries (Fig. **2**). These results suggested that the blockage of the ligand binding to the αVβ3 integrin may be an effective approach for the treatment of the proliferative phase of atherosclerosis in patients with diabetes [88].

Bempedoic acid, a medication in ongoing trials for the treatment of hypercholesterolaemia, targets hepatic ATP-citrate lyase, one of the enzymes involved in cholesterol biosynthesis [89]. Long-term treatment (160 days) of atherosclerotic Yucatan miniature pigs (*LDLR^+/−^* and *LDLR^−/−^*) with bempedoic acid decreased plasma cholesterol and LDL-C levels, while TG, HDL-C, fasting glucose and insulin, and liver lipids were not affected. Furthermore, the application of bempedoic acid reduced the lesion area in the aorta and coronary arteries, thus confirming its potential as an adjunct therapy for homozygous familial hypercholesterolaemia and atherosclerosis [90].

Pemafibrate, another potent drug for the treatment of metabolic and inflammatory diseases (such as hypertriglyceridaemia, non-alcoholic fatty liver disease, type 2 diabetes, and atherosclerosis), is an agonist of Peroxisome proliferator-activated receptor α (PPARα) - a major regulator of lipid metabolism in the liver [91, 92]. Administration of pemafibrate to *LDLR* knock-out pigs with balloon injury fed a high-fat diet resulted in reduced macrophage accumulation in the plaque area and decreased expression of *NF-κB*, *MMP9* and *Proto-Oncogene C-Jun* (*C-Jun*) genes. Interestingly, the intimal hyperplasia was not affected by pemafibrate. In total, pemafibrate may be considered a valuable medication for atherosclerosis treatment through plaque stabilisation and reduction of the inflammatory response [93].

##### 2.2.1.3 Natural bioactive compounds

Corilagin, a natural polyphenol with known anti-inflammatory and anti-tumour properties [94], has also demonstrated anti-atherosclerosis effects in minipigs atherosclerotic models. Administration of corilagin decreased the number of lipid plaques and foam cells and reduced the degree of injury in the common carotid artery. Moreover, the expression of *MMP-1/2/9* in common carotid artery plaques was downregulated by corilagin. Furthermore, *in vitro* experiments conducted on murine RAW264.7 macrophages revealed that corilagin inhibited nuclear translocation of NF-κB, thus suggesting wide-ranging anti-atherosclerotic therapeutic effects in both *in vivo* and *in vitro* conditions [95].

### 2.3 Zebrafish

Zebrafish, as a model system for hyperlipidaemia, atherosclerosis, and other cardiovascular diseases, has several advantages, such as compact size, high reproductive rate, ex utero fertilisation, embryonic transparency, swift development, conserved lipid metabolism, and a wide set of methods and tools available for experimental manipulability [96, 97]. Among the disadvantages of zebrafish as a model system to study atherosclerosis are the absence of unified protocols for experiments and husbandry practices and the limitation of high throughput screening of water-soluble compounds [98]. During recent years, several zebrafish models for hyperlipidaemia and atherosclerosis have been established, including a simple high-cholesterol diet-induced model and more advanced mutants of lipid metabolism-related genes (such as *LDLR* [99], *APOC2* [100], *Liver X receptor* (*LXR*) [101], *Apolipoprotein E* (*ApoEb*) [102], and *Apolipoprotein A-I binding protein* (*AIBP*) [103]. Detailed exploration of zebrafish atherosclerotic models was provided in several recent reviews [97, 104, 105]. Therefore, further in this section we discuss recently discovered natural compounds with anti-atherosclerosis effects tested on zebrafish atherosclerotic models.

Indole-3-carbinol (I3C) is a natural compound found in cruciferous vegetables and is well known for its anti-carcinogenic, antioxidant, and anti-inflammatory properties [106]. Administration of I3C to hyperlipidaemia zebrafish larvae fed a high-cholesterol diet (HCD) decreased lipid deposition of the vasculature in a dose-dependent manner, suggesting an anti-atherosclerotic effect (Fig. 2). Mechanically, I3C affected the expression of apoptosis and autophagy-related genes. In particular, the expression of *LC3-II*, *cathepsin D*, *beclin-1*, and *hVps34* was upregulated, while *AKT*, *P62*, *mTOR*, and *Bcl-2* were downregulated in zebrafish larvae fed with HCD [107]. Similarly, application of natural antioxidant Ethyl gallate, known as a potential treatment against diabetes and obesity [108], reduced vascular lipid accumulation and inflammatory responses in HCD fed zebrafish larvae [109].

Additionally, anti-atherogenic properties have been shown for some compounds derived from plants of *Dendrobium* genus. Thus, polysaccharides derived from *Dendrobium* *huoshanense* (C.Z.Tang et S.J.Cheng) improved the inflammatory response, oxidative stress, and lipid deposition parameters in HCD fed zebrafish. In particular, treatment with polysaccharides reduced TC, TG, MDA, and ROS levels, increased SOD activity and decreased neutrophil recruitment and plaque formation [110]. Water extracts of the related species *Dendrobium* *catenatum* (Lindl.) acted in a similar way, by reducing plaque formation, alleviating lipid metabolism disorder, oxidative stress, and inflammation response in an atherosclerotic zebrafish larval model [111]. These results suggested that bioactive compounds derived from plants of the *Dendrobium* genus have a high potential for future development of a natural treatment for atherosclerosis prevention.

### 2.4 Non-human Primates

Non-human primates exhibit hypercholesterolaemia and develop coronary fibro-fatty atherosclerotic lesions very similar to humans on a high-cholesterol/high-fat diet, thus offering substantial value in studying human atherosclerosis [112]. Subsequently, the application of genetic engineering methods allowed to create of more NHP mutants with accelerated atherosclerosis development or, vice versa, protected from atherosclerosis [37]. For example, the knock-out of lipoprotein (a) with the application of short interfering RNAs (siRNAs) in lean rhesus macaque monkeys allowed to study of the role of Lp(a) in atherosclerosis development and progression in primates [113].

Subsequently, a number of anti-atherosclerotic treatments have been investigated in non-human primate models. For example, inhibition of miR-33a/b, a repressor of the key cholesterol transporter ABCA1 and a major regulator of HDL biogenesis by anti-miR oligonucleotides in African green monkeys (*Chlorocebus aethiops*) increased plasma HDL levels and decreased levels of VLDL-associated triglycerides, thus inhibiting atherosclerosis development. Additionally, the application of miR-33 antagonist also increased the expression of other miR-33 target genes involved in the oxidation of fatty acids (such as *CROT*, *CPT1A*, *HADHB*, *PRKAA1*) and reduced genes involved in fatty acid synthesis (*SREBF1*, *FASN, ACLY*, *ACACA*), which greatly suppressed plasma VLDL TG levels. Notably, mice lack miR-33b, which was found only in the *SREBF1* gene of higher mammals, thus, mouse atherosclerosis models are not suitable for such investigation. In total, these data suggested that pharmacological inhibition of miR-33a/b is a promising therapeutic strategy for regulating plasma levels of HDL and VLDL for the treatment of metabolic and cardiovascular diseases [114].

The safety and efficacy of a novel HDL-based nano-immunotherapy were recently evaluated on cynomolgus monkeys (*Macaca fascicularis*). This nano-immunotherapy specifically blocked the CD40-TRAF6 interaction in monocytes and macrophages [115]. Cluster of differentiation (CD40) is an essential receptor mediating inflammatory and immune responses through interaction with different ligands [116, 117]. Tumour necrosis factor receptor-associated factor 6 (TRAF6) was shown to mediate signal transduction by CD40 signalling cascade inside monocytes and macrophages, while genetic deficiency or pharmacological inhibition of CD40-TRAF6 interaction impaired the recruitment of monocytes to the arterial wall, stimulated a switch in macrophage phenotype towards anti-inflammatory M2 type and reduced cardiac fibrosis and hypertrophy in mice model [118, 119]. Thus, the application of nano-immunotherapy demonstrated a reduction of plaque inflammation and favourable toxicity profiles in both mice and cynomolgus monkeys’ model systems, highlighting its high translation and therapeutic potential for the treatment of human atherosclerosis [115].

**Fig. (3):**
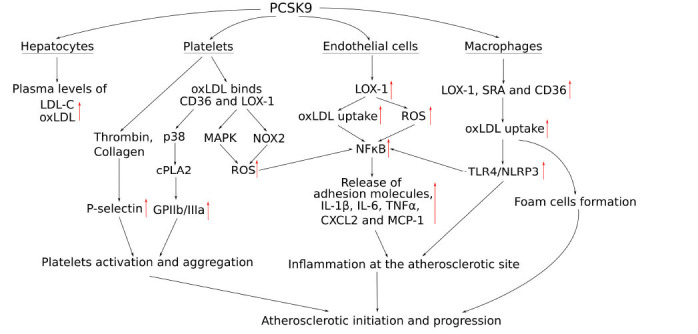
Schematic representation of the PCSK9 role in atherosclerosis development. PCSK9 upregulated the protein and expression levels of *LOX-1, CD36,* and *SRA*, thus increasing oxLDL uptake, ROS production, and promoting inflammation *via* NF-κB pathway activation. There are also some processes specific for a particular cell type: 1) In hepatocytes, the PCSK9–LDLR axis mainly causes the increased plasma levels of LDL and oxLDL; 2) the interaction between PCSK9 and CD36 activates cytosolic phospholipase A2 (cPLA2) via the p38MAPK pathway, which in further steps increase level of glycoprotein Iib/IIIa (GPIIb/IIIa). Moreover, PCSK9 and the binding of platelets with its agonists (such as collagen and thrombin) increase the level of P-selectin. Thus, by upregulating the levels of GPIIb/IIIa and P-selectin, PCSK9 facilitates platelet activation and adhesion. Furthermore, PCSK9/CD36 axis activates increased ROS production through MAPK and NOX2 pathways; 3) In endothelial cells, PCSK9 is crucial for the activation of inflammatory NF-κB pathways, the release of proinflammatory cytokines (such as IL-1β, IL-6 and TNFα), chemokines and adhesion molecules, which further promote plaque formation; 4) In macrophages, besides inflammatory NF-κB pathway activation, the increased oxLDL uptake promote foam cells formation and may also act through the toll-like receptor-4 (TLR4)/CD36 signalling pathway to activate NLR family pyrin domain containing 3 (NLRP3) inflammasome, which is also involved in the development of the vascular inflammation and atherosclerosis progression. Used abbreviations: PCSK9 - Proprotein convertase subtilisin/kexin type 9; LDL-C - low-density lipoprotein cholesterol; oxLDL - oxidised LDL; CD36 - cluster of differentiation 36; LOX-1 - lectin-like oxidised low-density lipoprotein receptor 1; p38 - p38 mitogen-activated protein kinase; cPLA2 - cytosolic phospholipase A2; GPIIb/IIIa - glycoprotein Iib/IIIa; NOX2 - NADPH oxidase type 2; ROS - reactive oxygen species; NF-κB - Nuclear factor kappa-light-chain-enhancer of activated B cells; IL - Interleukins; TNFα - Tumour necrosis factor alpha; CXCL2 - Chemokine (C-X-C motif) ligand 2; MCP-1 - monocyte chemoattractant protein-1; SRA - Macrophage scavenger receptor class A; TLR4 - toll-like receptor-4; NLRP3 - NLR family pyrin domain containing 3; LDL-R - low-density lipoprotein receptor. Red arrows represent PCSK9-mediated increased levels of corresponding gene expression, protein or biomolecules.

Proprotein convertase subtilisin/kexin type 9 (PCSK9) is a circulating hepatocytes’ produced protein that targets the degradation of the low-density lipoprotein receptor (LDLR) on the surface of hepatocytes. PCSK9 was shown as a crucial player in hypercholesterolaemia and atherosclerosis pathophysiology (Fig. **3**), while its inhibition decreased the level of LDL-C, reduced the inflammation response in the arterial wall and attenuated atherosclerotic plaque development [120]. Currently, inhibition of PCSK9 with human monoclonal antibodies is considered one of the most effective second-line treatments to lower LDL-C in adults with familial hypercholesterolaemia and atherosclerosis. Recently, several PCSK9 inhibiting immunotherapeutics have been approved for medical use in the USA and Europe (for example, alirocumab and evolocumab), while more are under investigation and are at different stages of trials [121-123].

CRISPR-based gene editing technologies have also been used to achieve the near-complete knockdown of *PCSK9* in the liver of cynomolgus monkeys with a single infusion of lipid nanoparticles as a delivery method. Animals receiving such treatment demonstrated a stable reduction of blood levels of PCSK9 and LDL-C for a period of at least 8 months after a single infusion (Fig. **2**). Notably, no adverse health events have been observed in any of the monkeys [124]. Similar results were obtained also with another CRISPR-based PCSK9-targeting therapy named VERVE-101. Accordingly, a single dose of VERVE-101 provided a stable reduction of the blood levels of PCSK9 and LDL-C for up to 476 days after treatment. Importantly, no germ-line transmission was detected in cynomolgus monkeys 3 months after treatment [125].

Another study has explored the stable PCSK9 knockdown in rhesus macaque (*Macaca mulatta*) liver with the application of adeno-associated virus (AAV)-delivered meganuclease as a gene delivery method. Treated monkeys showed sustained reductions in serum PCSK9 and LDL-C levels for a period of over 3 years. Moreover, no negative side effects were observed in the regular tests on blood samples, physical examination, ultrasound evaluations, and liver histopathology analyses [126].

Recently, the “Liposomal Immunogenic Fused PCSK9-Tetanus plus Alum adjuvant” (L-IFPTA) vaccine was developed with the use of a combination of the AFFITOPE^®^ and nanoliposome technologies [127]. The L-IFPTA vaccine stably produced antiPCSK9 antibodies and exerted long-lasting therapeutic and preventive effects in mice with severe hypercholesterolaemia and atherosclerosis [128]. Furthermore, the efficiency and safety of the L-IFPTA vaccine were evaluated in a preclinical study in healthy rhesus macaque monkeys. Immunised monkeys produced anti-PCSK9 antibodies, which caused no adverse effects on organ performance and plasma levels of pro- and anti-inflammatory markers, while the level of HDL-C was increased, thus suggesting that the L-IFPTA vaccine can be a good candidate for further clinical trials in humans [129].

In total, these studies demonstrated that the application of targeted *in vivo* immunotherapy, gene editing and knock-out methods can provide the required safety information and exert long-lasting therapeutic effects in non-human primates, thus having potential for clinical translation and can be beneficially applied in the treatment of cardiovascular diseases in humans.

## CONCLUSION

Animal models allow to conduct a fundamental research to identify and characterise the molecular mechanisms underlying the development and progression of atherosclerosis and to explore the potential applications of these mechanisms as drug targets. When selecting the best suitable animal model for the research of atherosclerosis or pharmaceutical medications for atherosclerosis treatment or prevention, various criteria (such as strain, age, diet and feeding period, circadian rhythm, and similarity to the desirable human pathophysiological situation) should be considered. In general, animal models of atherosclerosis require feeding on a special diet (such as high fat, cholesterol, or cholic acid) and/or genetic modification of genes involved in lipid metabolism (such as *Apo* family, *LDLR* or *CETP*). While wild-type animal models are absolutely required for any research, at least as a control group, most of the current research has been conducted on genetically modified animals.

Thus, although mice are considered the most popular, cheap, and widely used model species for atherosclerosis research with many mutants available, significant differences in lipid metabolism (such as, for example, the absence of CETP) and pathophysiology of disease progression prevent their wide use in the evaluation of the pharmacotherapies efficiency. Because the rabbit model of atherosclerosis better displays late-stage atherosclerosis and their body size allows certain surgical manipulation, it is often used in clinical research. As we have summarised, a rabbit model of atherosclerosis was used to elucidate the efficiency of novel stem/progenitor cell transplantation methods (such as human umbilical cord mesenchymal stem cells, rabbit adipose-derived stem cells and genetically modified endothelial progenitor cells) and repurposed anti-cancer (docetaxel, PJ34 and ruxolitinib) and anti-diabetic (semaglutide, gliclazide and dapagliflozin) drugs against atherosclerosis. However, the high breeding and handling prices and a limited number of available genetic models are serious drawbacks of rabbits as a model species for atherosclerosis research.

Moreover, while common pigs represent a good model of atherosclerosis research from the pathophysiological point of view, their use was greatly limited by a significant weight, associated with high costs of keeping and requirement for space. However, the invention of dwarf varieties and the application of genetic engineering methods greatly facilitated and expanded the use of pigs as a model system for atherosclerosis research. Thus, the anti-atherosclerosis efficiency of various repurposed drugs (such as spironolactone, bempedoic acid, and pemafibrate) was tested in mini-pig models. Zebrafish, another potent atherosclerosis model system, is ideal for quick and cheap screening of potential anti-atherosclerotic treatments. The major limitations of this system are: 1) the absence of unified experimental procedures and husbandry conditions; 2) only water-soluble compounds can be tested; 3) only early stages of atherosclerosis can be studied and 4) because of the small size of the specimen, specific imaging equipment should be used. As we summarised, the anti-atherogenic effect of several natural compounds has been recently tested in a zebrafish atherosclerosis model system – Indole-3-carbinol, Ethyl gallate, a polysaccharide derived from *D.* *huoshanense* and water extracts of *D.* *catenatum*.

Finally, non-human primates represent the best pathophysiological model of atherosclerosis. However, work with non-human primates is strictly regulated and extremely expensive. Moreover, experiments on NHP have raised significant ethical concerns, widely known as formulated in 50ss the 3Rs principles, which stands for Replacement (of animals by insentient material), Reduction (the number of animals used in experiments), and Refinement (decrease the severity of inhumane experimental procedures) [130]. Later the 3Rs principles were further expanded with concerns about animals’ housing and health conditions, application of appropriate anaesthesia or analgesia, and specific regulations for the creation of genetically modified animals [131]. Because of these, NHP are mostly used for the final stages of trials, when only a small number of the most promising drugs and treatments should be checked. Thus, we discuss the application of some advanced methods and techniques (such as immunotherapy, CRISP-based gene editing technologies, and adeno-associated virus-delivered meganuclease) to create anti-atherosclerotic treatment: HDL-based nano-immunotherapy and immunotherapeutic PCSK9 inhibition (VERVE-101 and L-IFPTA).

To date, no animal model perfectly replicates the complex properties of human atherosclerosis. Ideally, an animal model of atherosclerosis would develop lesions comparable to those in humans, as atherosclerotic plaques are the key clinically relevant pathophysiological feature of human atherosclerosis. Existing animal models are valuable for studying lesion development and testing therapeutic agents, yet anatomical, genetic, and lipid metabolism differences hinder the direct translation of findings to humans. In many animal models, hypercholesterolaemia is the primary factor driving lesion development, whereas, in humans, atherosclerosis is multifactorial. Additionally, murine models exhibit higher vascular inflammation compared to humans, making the targeting of inflammatory pathways more effective in mice but less so in human clinical settings. Another limitation is that in most animal models, atherosclerotic plaques form predominantly in the aorta and large proximal vessels, with rare occurrences in coronary vessels, unlike in humans. To better mimic human atherosclerosis and enhance translational potential, animal models with coronary artery plaque formation are needed. Moreover, the lack of plaque rupture, thrombosis, end-stage ischaemic lesions, myocardial infarction, and stroke in animal models is a significant shortcoming, as these events are common in human atherosclerosis.

Furthermore, the majority of atherosclerosis studies have been conducted on male animals, overlooking the gender-specific effects of the disease. Including both sexes in research, as recommended by the “NIH Policy on Sex as a Biological Variable,” would improve the confidence, rigour, and reproducibility of atherosclerosis studies. Overall, while existing animal models exhibit some characteristics of human plaques, they do not encompass all aspects. Developing new models that closely mimic human plaques, including features like neovascularisation, calcification, intra-plaque haemorrhage, and thrombosis, is critical. Such models would be instrumental in creating therapeutic agents for plaque stabilisation and enhancing scientific rigour.

Additionally, animal models of atherosclerotic plaque regression are needed to address the therapeutic goal of reversing pre-existing plaques. In patients, atherosclerosis often coexists with other conditions like diabetes and hypertension. Thus, complex animal models that incorporate these co-morbidities are essential for unravelling the mechanisms of atherosclerosis and developing safe, effective therapeutic agents for this prevalent human disease. In total, various animal models of atherosclerosis, including rabbit, zebrafish, pigs, and non-human primates, play a vital role in laboratory research, insightful translational studies of atherosclerosis and clinical testing of therapeutic interventions. In this review, we discuss several recent discoveries and the development of novel agents for the treatment and prevention of atherosclerosis and associated cardiovascular disease conducted on suitable non-rodent animal models.

## LIST OF ABBREVIATIONS

CVD: Cardiovascular Diseases
EC: Endothelial Cells
EPCS: Endothelial Progenitor Cells
FCH: Familial Combined Hyperlipidaemia
LDL: Low-Density Lipoprotein
MMP: Matrix Metalloproteinases
NHP: Non-Human Primates
ROS: Reactive Oxygen Species
SMC: Smooth Muscle Cells
TALEN: Transcription Activator-Like Effector Nuclease
VSMC: Vascular Smooth Muscle Cells
WHHL: Watanabe Heritable Hyperlipidemic
